# The magic of seaweed (*Ascophyllum nodosum*) extract in influencing the dynamics of yield, quality, and storage behavior of garlic

**DOI:** 10.3389/fpls.2025.1636319

**Published:** 2025-09-29

**Authors:** Aniket Mandal, Amit Baran Sharangi, Lamya Ahmed Al-Keridis, Safia Obaidur Rab, Nawaf Alshammari, Mohd Saeed, Mamdouh Alshammari, Nadiyah M. Alabdallahd

**Affiliations:** ^1^ Department of Plantation Spices Medicinal and Aromatic Crops, Bidhan Chandra Krishi Viswavidyalaya, Mohanpur, West Bengal, India; ^2^ Biology Department, Faculty of Science, Princess Nourah Bint Abdulrahman University, Riyadh, Saudi Arabia; ^3^ Department of Clinical Laboratory Sciences, College of Applied Medical Sciences, King Khalid University, Abha, Saudi Arabia; ^4^ Department of Biology, College of Science, University of Hail, Hail, Saudi Arabia; ^5^ Department of Biology, College of Science, Imam Abdulrahman Bin Faisal University, Dammam, Saudi Arabia; ^6^ Basic and Applied Scientific Research Centre, Imam Abdulrahman Bin Faisal University, Dammam, Saudi Arabia

**Keywords:** garlic, *Allium sativum* L., seaweed, *Ascophyllum nodosum*, growth, yield, quality

## Abstract

**Introduction:**

Since prehistoric times, garlic is one of the leading spices extensively cultivated for its flavour, pungency and medicinal values. Organo sulphur compounds viz., Allicin and diallyl disulfide are responsible for numerous medicinal properties. Among quite a few natural and synthetic substances, biostimulants have rewarding effects on garlic growth, stress tolerance, yield and productivity. *Ascophyllum nodosum* (seaweed) is such an inimitable biostimulant extensively used in garlic.

**Materials & Methods:**

The current study was conducted during two years at BCKV-Agricultural University, India to evaluate the effect of *A. nodosum*. Six different garlic genotypes including one local variety Goldana and 3 doses of *A. nodosum* were considered to study various morphological, yield attributing and quality parameters and their inter relationship with weather variables and the storage behaviour.

**Results and Discussion:**

The experiment revealed that AVT-1GNB-23-41 was the best among all the genotypes. The treatment AVT-1GNB-23-41×seaweed@1ml/L gave the maximum plant height (73.07cm),leaf width(2.32cm), polar & equatorial diameter (35.45, 36.33mm),clove length (2.64 cm), cloves/bulb (30.95), weight of bulb (18.87g), total yield (7.02t/ha) compared to others. AVT-1GNB-23-41 genotype showed highest TSS (30.83°Brix), phenol (159.65mg GAE/100g) while ascorbic acid (14.74mg/100g) was maximum in AVT-1GNB-23-26. In case of correlation study, bright sunshine hours and total rainfall had positive and negative correlations with total yield. L* and b* values decreased with advancement of storage but a* value was increased upto certain time and then decreased. On storage point of view, the treatment AVT-1GNB-23-47×seaweed@1ml/L was superior with lowest physiological weight loss at 36 DAST.

## 1 Introduction

Garlic (*Allium sativum* L.) is one of the most important of the 52 spices that are governed by the Spices Board under the Ministry of Commerce and Industry, Government of India. It has been extensively utilized in different cuisines since prehistoric times and is praised for its flavor and pungency. It comes under the family Alliaceae and originated from Central Asia (Bondre et al., 2017). After onion, it is considered as the second most extensively grown bulb crop. Approximately 75% of the world’s garlic is produced in China, with India coming in second (FAOSTAT, 2020). In India, the cultivation of garlic is broadly found in states such as Maharashtra, Madhya Pradesh, Gujarat, Uttar Pradesh, Orissa, Rajasthan, and West Bengal.

**Figure 1:**
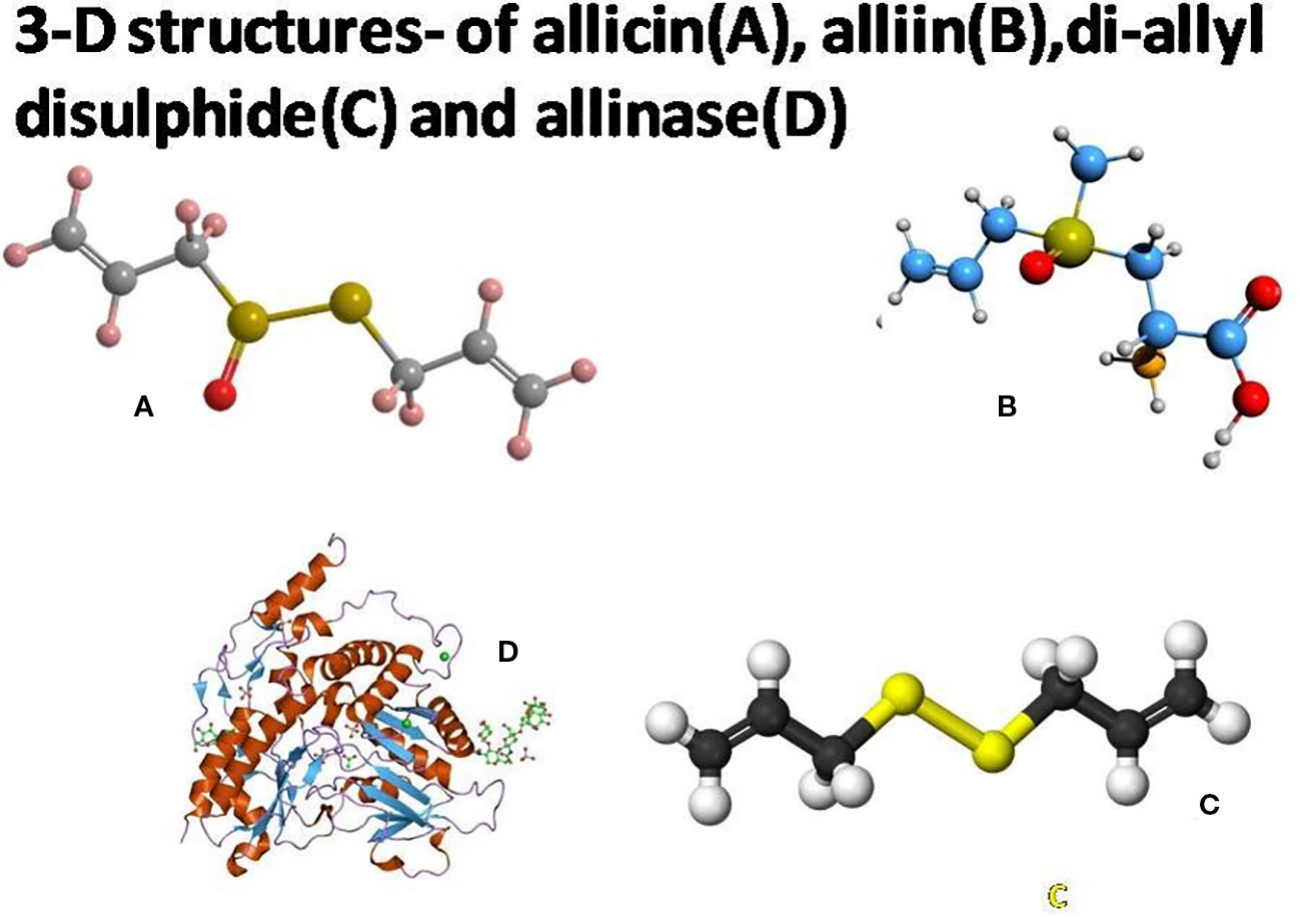
Three-dimensional structures of allicin **(A)**, alliin **(B)**, diallyl disulfide **(C)**, and alliinase **(D)**. Source: Wikipedia, 2025; Molview, 2025.

Garlic is not only used to flavor foods, but its allicin molecules also have remarkable medicinal value. Diallyl disulfide is an important organosulfur substance present in garlic (Song et al., 2021). This compound is formed as a result of the decomposition of allicin (Lanzotti, 2006) (**Figure 1**). Raw garlic bulbs (fresh) consist of ~65.88% water, ~1.28% amino acids, ~1.54% fiber, ~2.45% protein, ~26.79% carbohydrate, phenols, fatty acids, and trace compounds, and more than 33.81 (~2.37%) sulfur-containing molecules (Butt et al., 2009). Garlic appears to enhance the functioning of the immune system by stimulating certain cell types. The oil comprises antioxidant substances that play pivotal roles in the fight against harmful free radicals. Fresh garlic improves the digestive and reproductive activity of the human body (Lee et al., 2015; Jafari et al., 2023). It helps to lower high blood pressure and improves the blood circulation system (Cicero and Borghi, 2013). Furthermore, it has anti-anemic, anti-atherogenic, anti-hypertensive, anti-carcinogenic, antitumor, antimicrobial, antifungal, and immunomodulatory properties. Garlic has a chemical element called selenium (Se), which is responsible for lowering the cholesterol level. It hinders the progress of coronary calcification (Weiss et al., 2013; Li et al., 2022). Organic sulfur compounds have antiviral properties. These compounds damage viral receptors or inhibit the synthesis mechanisms of viral nucleic acids, in addition to protecting against these viruses.

Biostimulants are substances that occur naturally or by synthetic means. According to Franzoni et al. (2022), biostimulants are compounds, whether organic or inorganic, that contain bioactive agents or microorganisms. When introduced into plants or their root zones, they promote plant growth and productivity by enhancing nutrient uptake and utilization. In addition, these substances improve the ability of plants to withstand environmental stresses and enhance the quality of their output, regardless of their nutrient content. These elements have beneficial effects on plant growth and development, abiotic stress resistance/tolerance, crop yield, and productivity. With the goal of promoting growth, reducing stress-induced constraints, and increasing yields, biostimulants present a potentially revolutionary method for controlling and/or altering physiological processes in plants. A combination of synthetic and natural chemicals made of hormones or plant hormone precursors is typically used to produce biostimulants. Its correct application in crops has the potential to positively impact growth and development by directly affecting physiological processes. Biostimulants can be grouped into five different categories: i) seaweed extracts; ii) hydrolyzed protein- and nitrogen-based components; iii) humic substances; iv) inorganic compounds with biostimulant action; and v) microorganisms. Seaweeds come under a large group from multicellular to microscopic marine algae associated with various taxonomic groups including red algae, brown algae, and green algae. In ancient times, algae have been used as fertilizers. At present, seaweed extract is used for soil conditioning or as a biostimulant. Foliar treatment is the best method to utilize the full efficiency of seaweed extract. As a result, maximum growth, crop yield, and abiotic stress resistance are achieved.

Among the various groups of seaweeds, *Ascophyllum nodosum*, which belongs to the Phaeophyta group, has been the subject of most research. It is a source of numerous industrial and commercial biostimulators that are used to strengthen the plant defense mechanism against biotic and abiotic challenges by better regulating physicochemical processes (Shukla et al., 2019). *A. nodosum*, sometimes referred to as knotted kelp, rockweed, or Norwegian kelp, among other names, is edible brown algae. It is mostly found in the North Atlantic Ocean, which reaches from eastern Canada to portions of Northern Europe (Cardozo et al., 2007).

Seaweed extract from *A. nodosum* contains plant growth regulators, including auxins, betaines, gibberellins, cytokinins, organic acids, polysaccharides, and amino acids, which could enhance plant growth, yield, and quality. Phlorotannins are special polyphenolic chemicals that are not present in terrestrial plants and are only present in some brown algae. *A. nodosum* is one of the most important sources of phlorotannins (Connan et al., 2004; Sardari et al., 2021). In addition to its non-polysaccharide bioactive components including polyphenols, *A. nodosum* contains bioactive biopolymers such as fucoidan, laminarin, and alginates (Chater, 2016). Commercial *A. nodosum* extracts can be found on the market under a number of different trade names, including AgriGro Ultra, Maxicrop, Biovita, Acadian, Alg-A-Mic, Nitrozime, Bio-Genesis, High Tide, Guarantee, Espoma, Kelp Meal, Stimplex, and Synergy, among others (Ali et al., 2019a, 2019b).

Shalaby and El-Ramady (2014) reported that the total yield and the quality of the bulb in garlic improved with foliar treatment of biostimulants, including yeast (2 g/l) and amino total (1.2 ml/L). Under sandy soil conditions, garlic plants sprayed with humic acid at 0.2% and green microalgae extract at 0.2% exhibited an increment in the mineral uptake, growth rate, and total yield of plants (Abou El-Khair et al., 2010). Seaweed has been found to be beneficial for garlic (Fawzy et al., 2012). The bulb weight, nitrogen concentration, total yield, and crude protein percentage in the dry matter of the bulb of green garlic were highest when administered foliar spray of *A. nodosum* extract at 1 ml/L (Osman et al., 2021).

Garlic cultivation is influenced by temperature fluctuations across its growth phases. Cold temperatures during bulb development can retard maturity and result in a diminished bulb size. On the contrary, excessively high temperatures during bulb formation may hamper proper bulb development, leading to smaller cloves. Moreover, overabundant rainfall or irrigation can induce waterlogging, promoting bulb rot and, consequently, reducing the yield. Fluctuations in the humidity levels beyond optimal ranges can also detrimentally affect the bulb yield. Furthermore, adequate sunlight is imperative for facilitating photosynthesis and promoting robust bulb development in garlic crops.

The quality of garlic bulbs can significantly decline due to unfavorable storage conditions over extended periods (Volk and Rotindo, 2004). Throughout storage, issues such as sprouting, rooting, weight reduction, discoloration, undesirable flavor, and increased microbial activity contribute to loss of quality. Commercial storage of garlic bulbs and cloves typically occurs in either warehouses or conventional cold storage facilities. Warehouses tend to have higher ambient temperatures and lower relative humidity compared with regular cold stores. Consequently, the total product loss during prolonged storage can range from 25% to 40%. Therefore, there should be some standard methods for long-term storage of garlic.

A good number of studies have been conducted in the past to examine how various biostimulants affect the yield and quality metrics of garlic (Ali, 2017; Balmori et al., 2019; Abou El-Khair et al., 2010; Hamam et al., 2021; Dawa et al., 2012). However, there is a dearth of information on the effects of *A. nodosum* seaweed extract, specifically with regard to the garlic production in West Bengal. Along with this, it has been observed that there is limited research conducted on the storage behavior of garlic bulbs and the influence of various weather parameters on the yield and quality of garlic under the conditions prevalent in West Bengal. Keeping this in consideration, the present study was carried out to determine the effect of *A. nodosum* on yield, quality, and storage behavior at room temperature and to study the environmental impact on the yield of garlic.

## 2 Materials and methods

### 2.1 Details of the experimental site

**Figure 2:**
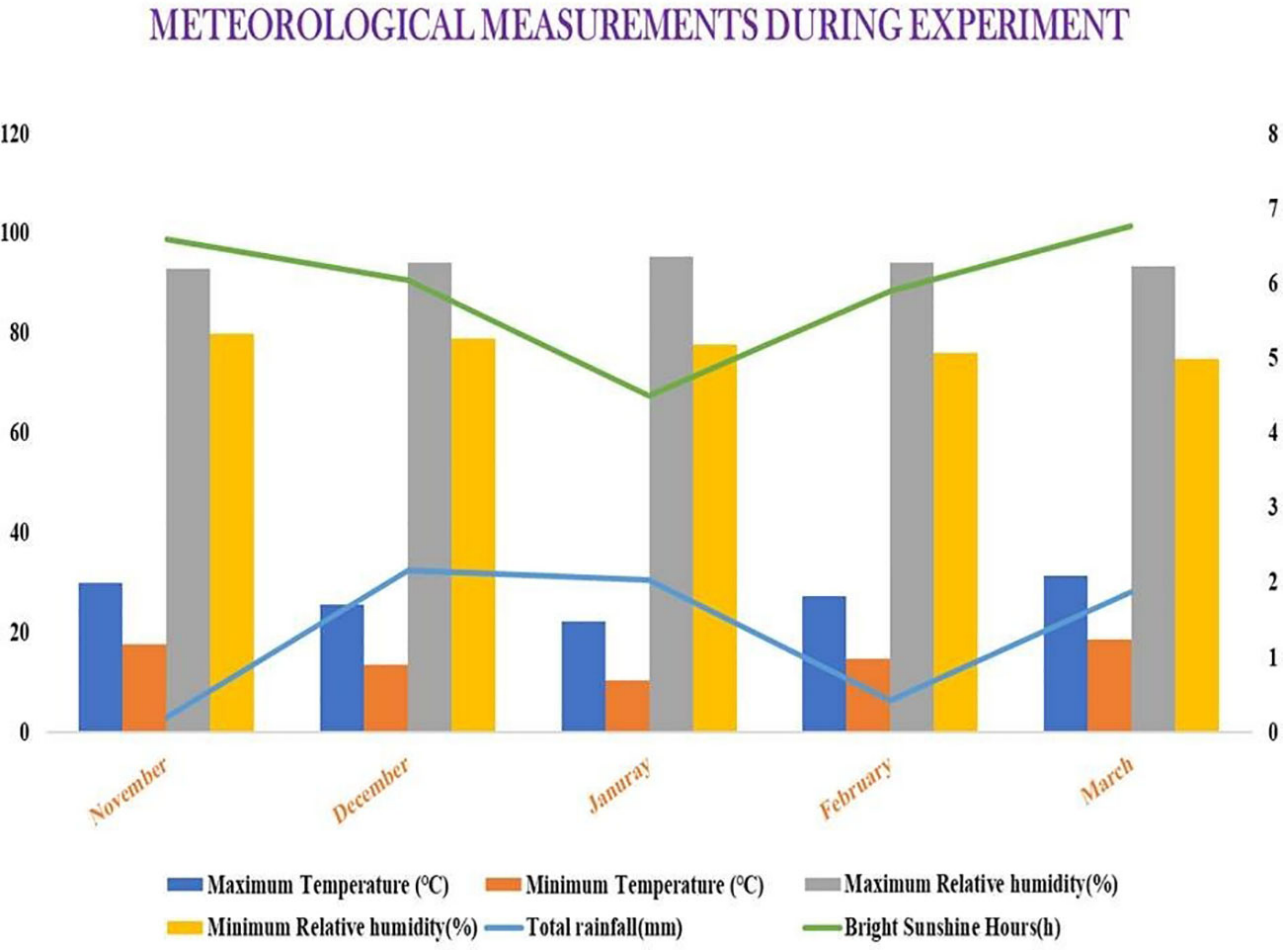
Weather parameters during the experimental period (from November to March). Source: Department of Agricultural Meteorology and Physics, BCKV, West Bengal.

The present experiment was conducted at the recognized research plots of AINRP on Onion and Garlic (ICAR), located at BCKV, Kalyani, India, situated at coordinates 22°C57′ N latitude and 88°C20′ E longitude, with an average altitude of 9.75 m above mean sea level (**Supplementary Figure S1**). The average temperature ranges from 25°CC to 36.5°CC during the summer months and between 12°CC and 25°CC during the winter months. Typically, the monsoon season commences on the second fortnight of June and persists until September. Occasional light rainfall can be expected during the winter season. This location experiences a warm subtropical humid climate within the torrid regions, but benefits from its proximity to the Bay of Bengal and a network of rivers, which mitigate the extreme conditions. The experimental site features a Gangetic alluvial soil (Entisol) with a sandy clay loam structure, characterized by good water holding capacity and proper drainage, along with moderate soil fertility. The physicochemical attributes of the soil at depths of 0–25 cm showed pH, electrical conductivity (EC), and organic carbon of 7.17, 0.23 dS/m, and 0.55%, respectively, along with available N, P, and K contents of 208.56, 8.04, and 184.97 kg/ha, respectively (**Supplementary Table S1**). The weather parameters, namely, temperature, rainfall, relative humidity, and bright sunshine hours, during the experimental period are depicted in **Figure 2**.

### 2.2 Treatments, experimental design, and other details

Five different genotypes—AVT-1 GNB-23-38, AVT-1 GNB-23-41, AVT-1 GNB-23-47, AVT-1 GNB-23-20, and AVT-1 GNB-23-26—were collected from the National Horticultural Research and Development Foundation, Nasik, and one local variety (Goldana) was purchased from the Kalyani market (**Supplementary Table S2**). Biovita, a commercial seaweed extract obtained from *A. nodosum*, was purchased from a local agrochemical shop in Kalyani, India. The experiment used a factorial randomized block design (RBD) with 16 treatments replicated three times (each of the five varieties were treated with *A. nodosum* at doses of 0.5, 1.0, and 1.5 ml/L, thereby comprising 15 treatments plus one local variety with no spray treatment serving as the control). The plot size and the spacing were 2 m × 1.95 m and 15 cm × 10 cm, respectively. The crop was sown on November 10, 2023, and was harvested on March 29, 2024. Along with the application of farmyard manure at 25 t/ha, nitrogen (N), phosphorus (P), and potassium (K) were administered, adhering to the recommended dose of 100:50:50 kg NPK per hectare (DOGR-ICAR, 2024). All intercultural operations, including irrigation, weeding, and plant protection measures, were performed as and when required. Plants were individually uprooted, cleaned, bundled, and cured on sand under shade for a period of 15 days.

### 2.3 Observations recorded

#### 2.3.1 Biometric observation

Measurements of the growth parameters of garlic, i.e., plant height, number of leaves/plant, neck thickness, leaf length, and leaf width, among others, were recorded. Five plants from each plot within every replication were randomly selected, and the average data were subjected to statistical analysis. Plant height was measured from the base of the plant to the tip of the stem, while the number of branches was counted per plant.

#### 2.3.2 Yield and yield attributing parameters

For the polar and equatorial diameters (in millimeters) of garlic cloves, measurements were taken from five randomly selected bulbs, with averages computed. The average bulb weight was measured using a digital weighing machine, with averages calculated in grams. The clove length and clove width of five randomly chosen cloves from bulbs were measured using Vernier callipers, with averages calculated in centimeters. For the number of cloves/bulb, the count was determined from five randomly selected bulbs and averaged. The bulbs from each plot were harvested and weighed using a digital weighing machine, with the measurements recorded in kilograms. After curing and leaf trimming (2–2.5 cm above the neck), the bulbs from each plot were again weighed using a digital weighing machine, with measurements recorded in kilograms.

#### 2.3.3 Quality analysis

Quality analyses on the total soluble solids (TSS), ascorbic acid, total sugar, reducing sugar, non-reducing sugar, and total phenol were performed in garlic after harvest. Garlic cloves were crushed using a pestle and mortar, and then the juice was extracted by pressing them through a muslin cloth. One drop of this juice was placed onto a hand refractometer. Data were collected and the average value from all five bulbs was determined. To estimate the ascorbic acid content in garlic, titration against 2,6-dichlorophenol-indophenol dye was conducted following the method described by Ranganna (2001). The results were expressed as milligrams per 100 g of bulb. The total, reducing, and non-reducing sugars were estimated using Fehling’s solutions with conventional anthrone reagent methods. The concentration of total phenolics expressed as GAE (Gallic acid equivalents) was determined using a modified version of the method outlined by Singleton and Rossi (1965).

The color/chromaticity values, i.e., L*, a*, b*, chroma (*C*) (Equation 1), the HUE angle (*H*) (Equation 2), and the browning index (BI) (Equation 3), were obtained using a Hunter colorimeter (Hunter Associates Laboratory, Inc., HunterLab, a US-based company in India), specifically Color Quest XE, which functions as a dual-beam xenon flash spectrophotometer with a spectral range spanning from 400 to 700 nm. Physiological loss in weight of the bulb was also taken. The L* value represents luminance or brightness. The a* value denotes the position on the red–green axis, with positive values indicating a tendency toward red–purple hues and negative values indicating a tendency toward green. Conversely, the b* value signifies the position on the yellow–blue axis, where positive values suggest a leaning toward yellow and negative values indicate a tendency toward blue. Chroma indicates the degree to which a color appears pure or vivid and is directly correlated with the color’s strength or richness. A hue angle between 0°C and 360°C typically corresponds to a red hue, while angles of 90°C, 180°C, and 270°C represent yellow, green, and blue hues, respectively. The browning index is linked to the intensity of brown coloration (Ding et al., 2020).


(1) $$\text{C}=\sqrt{a^{2}+b^{2}}$$

(2) $$\text{H}=\tan^{-1}(\frac{b}{a})$$

(3) $$\text{BI}=\frac{\left[100(X-0.31)\right]}{0.17}$$

where $\text{x}=\frac{\left(a+1.75L\right)}{5.645L+a-0.3012b}$ .

Growing degree days (GDD) is a metric that measures how much plants grow and develop throughout the growing season. The daily maximum and minimum temperatures are given as inputs to compute the GDD (Equation 4).


(4) $$GDD=\frac{T_{max}+T_{min}}{2}-T_{base}$$

Here, *T*_max_ is the daily maximum temperature, *T*_min_ is the daily minimum temperature, and *T*_base_ is the base temperature (for garlic, 5°C). Below this temperature, plant growth will not occur (El-Zohiri and Farag, 2014).

#### 2.3.4 Storage studies

The physiological weight loss of bulbs was monitored at 6, 12, 18, 24, 30, and 36 days after storage (DAST) using an electronic balance. The cumulative loss in weight was computed and presented as the physiological weight loss, according to the following formula.


$$\text{PLW}(\%)=\frac{{\text{P}}_{0}-{\text{P}}_{1}\text{ or }{\text{P}}_{2}\text{ or }{\text{P}}_{3}\text{ or }{\text{P}}_{3}\text{ or }{\text{P}}_{4}\text{ or }{\text{P}}_{5}\text{ or }{\text{P}}_{6}}{{\text{P}}_{0}}\times 100$$

where *P*_0_ indicates the initial weight, *P*_1_ is the weight after 6 days, *P*_2_ the weight after 12 days, *P*_3_ the weight after 18 days, *P*_4_ the weight after 24 days, *P*_5_ the weight after 30 days, and *P*_6_ is the weight after 36 days.

#### 2.3.5 Statistical analysis

Quantitative data were analyzed using analysis of variance (ANOVA), followed by subsequent application of Duncan’s multiple range test after confirming the normality and homogeneity of variance (IBM SPSS, v.25.0). Mean comparisons were conducted using the least significant difference (LSD) test with a significance level of *p* = 0.05. Correlation analysis was performed using Pearson’s correlation coefficient at *p* = 0.001, 0.01, and 0.05 levels, and correlograms were drawn with the software GRAPES (General R-shiny based Analysis Platform Empowered by Statistics, v.1.1.0).

## 3 Results

The results of the impact of seaweed and genotypes of garlic on various morphological, yield attributing, and quality parameters, along with their inter-relationships with the weather variables and the storage behavior, have been illustrated in a previous chapter. However, the salient observations of the same are summarized below.

### 3.1 Morphological, yield attributing, and yield characteristics

**Figure 3:**
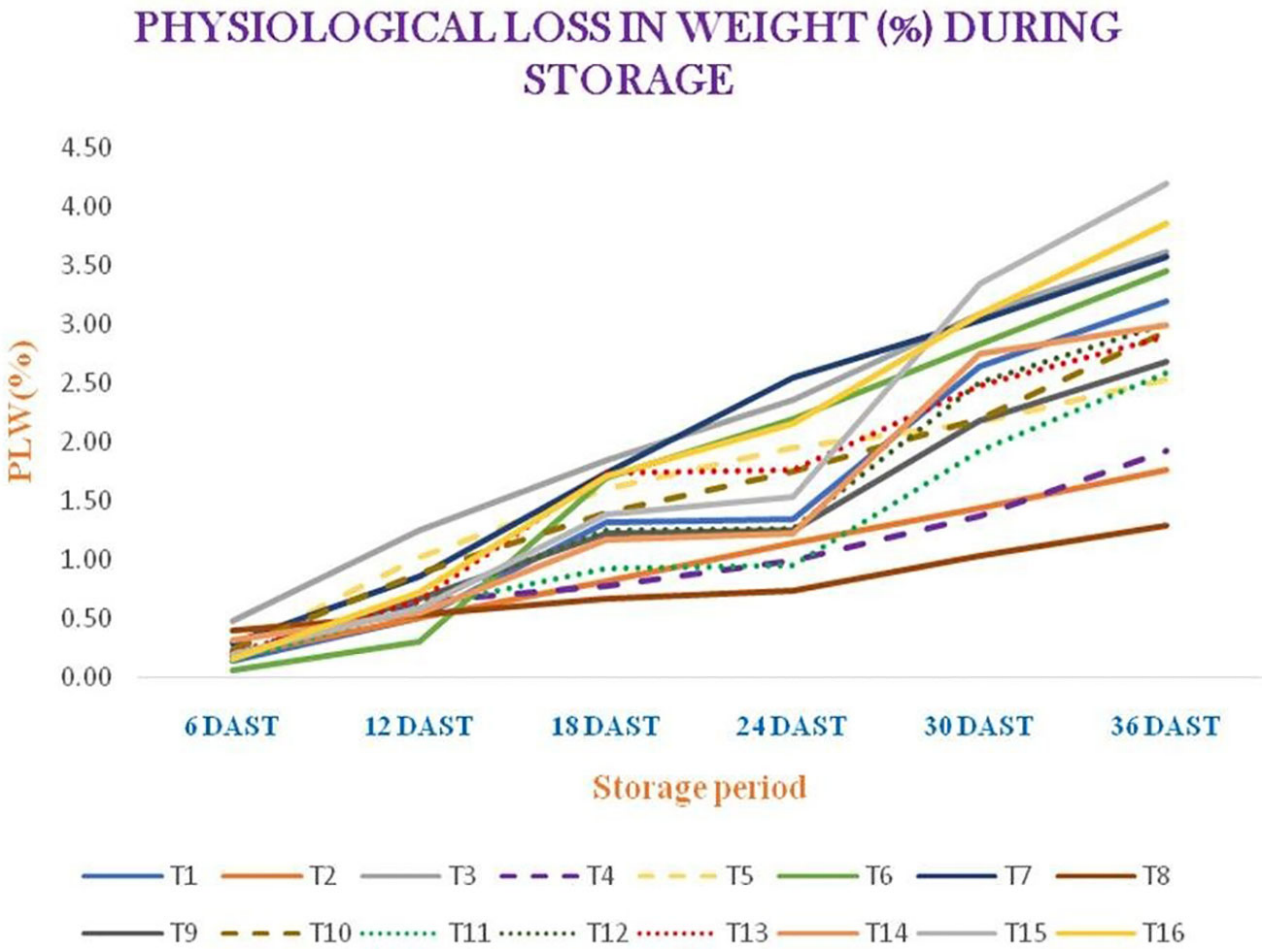
Total yield (in tons per hectare) as influenced by the interaction genotype × *Ascophyllum nodosum*.

Different morphological, yield attributing, and yield parameters, including plant height (in centimeters), number of leaves per plant, leaf length (in centimeters), leaf width (in centimeters), neck thickness (in millimeters), polar diameter (in millimeters), equatorial diameter (in millimeters), clove width (in centimeters), clove length (in centimeters), number of cloves per bulb, average weight of bulb (in grams), total yield (in kilograms per 3.9 m^2^), marketable yield (in kilograms per 3.9 m^2^), total yield (in tons per hectare), marketable yield (in tons per hectare), and heat use efficiency of garlic, were taken into consideration, the majority of which are presented in **Supplementary Tables S3** and **S4**. The plant height at the time of harvest, i.e., 140 DAST, was highest in genotype AVT-1 GNB-23-26 (71.14 cm) compared with the local variety, Goldana (62.93 cm). Maximum number of leaves per plant was observed in AVT-1 GNB-23-41 (7.07) compared with the local variety (5.97). Maximum leaf length was observed in genotype AVT-1 GNB-23-47 (44.76 cm) compared with the local variety (40.00 cm). Furthermore, the highest leaf width was observed in genotype AVT-1 GNB-23-41 (2.11 cm) compared with the local variety (1.67 cm). The neck thickness of genotype AVT-1 GNB-23-20 (7.30 mm) was greater compared with that of the local variety (6.01 mm). Maximum polar diameter was observed in genotype AVT-1 GNB-23-41 (34.32 mm) compared with the local variety (24.84 mm). The equatorial diameter in genotype AVT-1 GNB-23-41 (35.12 mm) was greater compared with the local variety (29.08 mm). In addition, clove width was observed highest in genotype AVT-1 GNB-23-41 (1.12 cm) compared with the local variety (0.67 cm). Maximum clove length was observed in genotype AVT-1 GNB-23-41 (2.59 cm) compared with the local variety (2.30 cm). The number of cloves per bulb was observed highest in genotype AVT-1 GNB-23-41 (29.72) compared with the local variety (23.90). The average weight of bulb in genotype AVT-1 GNB-23-41 (18.32 g) was greater compared with the local variety (13.56 g). Maximum total yield was observed in genotype AVT-1 GNB-23-41 (2.62 kg/3.9 m^2^) compared with the local variety (2.16 kg/3.9 m^2^), and 21.29% more total yield was observed in genotype AVT-1 GNB-23-41 compared with the local variety. The highest marketable yield was observed in genotype AVT-1 GNB-23-41 (2.57 kg/3.9 m^2^) compared with the local variety (2.11 kg/3.9 m^2^). Maximum total yield was observed in genotype AVT-1 GNB-23-41 (6.55 t/ha) compared with the local variety (5.39 t/ha) (**Figure 3**). The marketable yield in genotype AVT-1 GNB-23-41 (6.42 t/ha) was greater compared with the local variety (5.27 t/ha). Maximum heat use efficiency was found in AVT-1 GNB-23-41 + *A. nodosum* at 1 ml/L (3.390 kg ha^−1^ day^−1^), while local variety + no spray exhibited the lowest heat use efficiency (2.606 kg ha^−1^ day^−1^).

**Figure 4:**
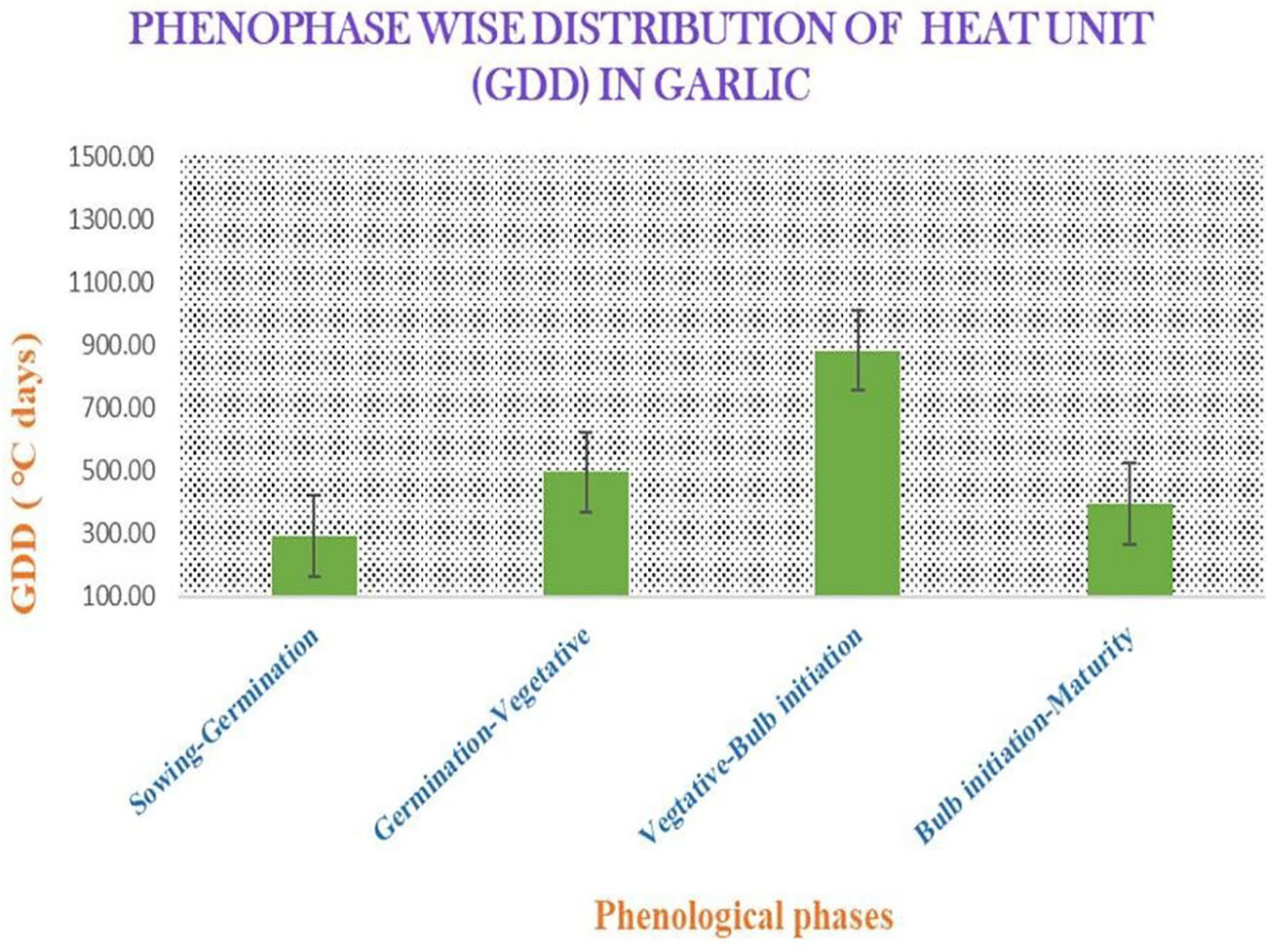
Phenophase-wise distribution of the heat units (growing degree days, GDD) in garlic.

### 3.2 Quality attributes

Various quality parameters of garlic, including TSS (°CBrix), ascorbic acid (in milligrams per 100 g), total phenol (in milligrams GAE/100 g), total sugar (in percent), reducing sugar (in percent), and non-reducing sugar (in percent), were studied. Maximum TSS content was observed in genotype AVT-1 GNB-23-41 (30.83°CBrix) compared with the local variety (27.35°CBrix). In the case of interaction, AVT-1 GNB-23-41 × *A. nodosum* at 0.5 ml/L gave the maximum TSS content (31.10°CBrix), while the minimum TSS content was observed in AVT-1 GNB-23-20 × *A. nodosum* at 1.5 ml/L (27.92°CBrix). The ascorbic acid content in genotype AVT-1 GNB-23-26 (14.74 mg/100 g) was greater compared with the local variety (12.86 mg/100 g). Furthermore, AVT-1 GNB-23-26 × *A. nodosum* at 1 ml/L had the highest ascorbic acid content (15.87 mg/100 g), while the lowest ascorbic acid content was determined in AVT-1 GNB-23-47 × *A. nodosum* at 0.5 ml/L (13.37 mg/100 g). The total phenol content was observed highest in genotype AVT-1 GNB-23-41 (159.65 mg GAE/100 g) compared with the local variety (50.83 mg GAE/100 g). In the case of interaction effect, AVT-1 GNB-23-41 × *A. nodosum* at 1 ml/L gave the maximum total phenol content (161.09 mg GAE/100 g), followed by AVT-1 GNB-23-41 × *A. nodosum* at 1.5 ml/L (159.75 mg GAE/100 g), whereas the minimum total phenol content was obtained in AVT-1 GNB-23-38 × *A. nodosum* at 1.5 ml/L (152.41 mg GAE/100 g). Maximum total sugar was observed in genotype AVT-1 GNB-23-38 (5.93%) compared with the local variety (4.27%). Moreover, AVT-1 GNB-23-38 × *A. nodosum* at 1 ml/L gave the highest total sugar (6.25%), while the lowest value was determined in AVT-1 GNB-23-41 × *A. nodosum* at 1.5 ml/L (4.39%). Reducing sugar was observed highest in genotype AVT-1 GNB-23-20 (2.97%) compared with the local variety (1.96%). In addition, AVT-1 GNB-23-20 × *A. nodosum* at 0.5 ml/L gave the maximum reducing sugar (3.13%), whereas the minimum reducing sugar was obtained in AVT-1 GNB-23-41 × *A. nodosum* at 1.5 ml/L (1.98%). The amount of non-reducing sugar in genotype AVT-1 GNB-23-38 (3.31%) was greater compared with the local variety (2.31%). Furthermore, AVT-1 GNB-23-38 × *A. nodosum* at 1 ml/L gave the highest non-reducing sugar (3.42%), while the lowest non-reducing sugar was determined in AVT-1 GNB-23-26 × *A. nodosum* at 1.5 ml/L (2.17%).

### 3.3 Inter-relationships between the morphological, yield, quality, and weather parameters

Total yield demonstrated a highly significant and positive correlation with plant height (0.756), polar diameter (0.862), and equatorial diameter (0.816). Total yield was also significantly correlated with the number of cloves per bulb (0.888). There was a positive and significant correlation between total yield and clove length (0.922) and clove width (0.917). Total yield exhibited a significant correlation with the average weight of bulb (0.492). The average weight of bulb was significantly correlated with plant height (0.568), leaf length (0.629), polar diameter (0.474), number of cloves per bulb (0.467), clove length (0.658), and clove width (0.553). There was a positive and significant correlation between total yield and TSS content (0.54). Total yield also demonstrated a positive and significant correlation with the total phenol content (0.526). Interestingly, non-reducing sugar (−0.565) was significantly correlated with total yield, but it was a negative association. Total yield had a significant and negative correlation with total rainfall (−0.581). A significant and positive correlation was found between total yield and bright sunshine hours (0.471) (**Table 1**).

**Table 1:** Correlation coefficient analysis between the weather parameters and the yield of garlic.

Characteristic	*T*_max_	*T*_min_	RH I	RH II	TR	BSH	TY
*T*_max_	1						
*T*_min_	0.82^a^	1					
RH I	−0.419	−0.306	1				
RH II	−0.416	−0.097	0.376	1			
TR	0.038	0.213	0.134	0.274	1		
BSH	0.527^b^	0.037	−0.42	−0.486^b^	−0.163	1	
TY	0.251	0.024	−0.043	−0.4	−0.581^c^	0.471^b^	1

*T*_max_, maximum temperature (in degree Celsius); *T*_min_, minimum temperature (in degree Celsius); *RH I*, morning relative humidity (in percent); *RH II*, evening relative humidity (in percent); *TR*, total rainfall (in millimeters); *BSH*, bright sunshine hours; *TY*, total yield (in tons per hectare).

a Correlation is significant at the 0.001 level.

b Correlation is significant at the 0.05 level.

c Correlation is significant at the 0.01 level.

### 3.4 Storage studies

**Figure 5:**
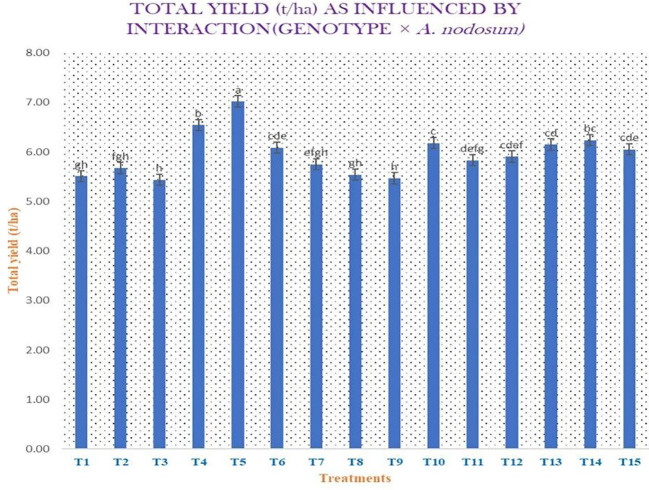
Physiological weight loss (in percent) during storage. *DAST*, days after storage. T_1_ = AVT-1 GNB-23-38 + *Ascophyllum nodosum* at 0.5 ml/L; T_2_ = AVT-1 GNB-23-38 + *A. nodosum* at 1 ml/L;T_3_ = AVT-1 GNB-23-38 + *A. nodosum* at 1.5 ml/L; T_4_ = AVT-1 GNB-23-41 + *A. nodosum* at 0.5 ml/L; T_5_ = AVT-1 GNB-23-41 + *A. nodosum* at 1 ml/L;T_6_ = AVT-1 GNB-23-41 + *A. nodosum* at 1.5 ml/L; T_7_ = AVT-1 GNB-23-47 + *A. nodosum* at 0.5 ml/L; T_8_ = AVT-1 GNB-23-47 + *A. nodosum* at 1 ml/L; T_9_ = AVT-1 GNB-23-47 + *A. nodosum* at 1.5 ml/L; T_10_ = AVT-1 GNB-23-20 + *A. nodosum* at 0.5 ml/L; T_11_ = AVT-1 GNB-23-20 + *A. nodosum* at 1 ml/L; T_12_ = AVT-1 GNB-23-20 + *A. nodosum* at 1.5 ml/L; T_13_ = AVT-1 GNB-23-26 + *A. nodosum* at 0.5 ml/L; T_14_ = AVT-1 GNB-23-26 + *A. nodosum* at 1 ml/L; T_15_ = AVT-1 GNB-23-26 + *A. nodosum* at 1.5 ml/L; T_16_ = local variety + no spray. Values with same letter(s) within a column are not significantly different at P= 0.05 (Duncun's Multiple Range Test, DMRT).

Different storage-related observations, such as the color values (i.e., L*, a*, and b*), chroma, hue angle (in degrees), the browning index, and the physiological weight loss (in percent) of garlic, were taken into consideration in relation to various treatments: T_1_ (AVT-1 GNB-23-38 + *A. nodosum* at 0.5 ml/L), T_2_ (AVT-1 GNB-23-38 + *A. nodosum* at 1 ml/L), T_3_ (AVT-1 GNB-23-38 + *A. nodosum* at 1.5 ml/L), T_4_ (AVT-1 GNB-23-41 + *A. nodosum* at 0.5 ml/L), T_5_ (AVT-1 GNB-23-41 + *A. nodosum* at 1 ml/L), T_6_ (AVT-1 GNB-23-41 + *A. nodosum* at 1.5 ml/L), T_7_ (AVT-1 GNB-23-47 + *A. nodosum* at 0.5 ml/L), T_8_ (AVT-1 GNB-23-47 + *A. nodosum* at 1 ml/L), T_9_ (AVT-1 GNB-23-47 + *A. nodosum* at 1.5 ml/L), T_10_ (AVT-1 GNB-23-20 + *A. nodosum* at 0.5 ml/L), T_11_ (AVT-1 GNB-23-20 + *A. nodosum* at 1 ml/L), T_12_ (AVT-1 GNB-23-20 + *A. nodosum* at 1.5 ml/L), T_13_ (AVT-1 GNB-23-26 + *A. nodosum* at 0.5 ml/L), T_14_ (AVT-1 GNB-23-26 + *A. nodosum* at 1 ml/L), T_15_ (AVT-1 GNB-23-26 + *A. nodosum* at 1.5 ml/L), and T_16_ (local variety + no spray). A decreasing trend in the L* value was observed in the storage experiment. T_11_ demonstrated the highest L* value (38.42) at 36 DAST, whereas the lowest L* value was observed in T_2_ (29.47) for the whole garlic bulb. Moreover, in the case of garlic paste, T_8_ and T_10_ gave the maximum L* value (52.73), followed by T_7_ (52.34), at 36 DAST, while the lowest L* value was observed in T_1_ (47.50), followed by T_3_ (48.03) (**Supplementary Table S3**). The a* value increased up to 12 DAST, then showed a decreasing trend in the case of whole garlic bulb. However, in the case of garlic paste, the value initially increased, but a decreasing trend was noted at 24 DAST. T_8_ demonstrated the highest a* value (1.89) at 36 DAST, whereas the lowest a* value was observed in T_15_ (−1.67) for whole garlic bulb. Moreover, for garlic paste, T_4_ gave the maximum a* value (2.23) at 36 DAST, while the minimum a* value was observed in T_12_ (0.55), followed by T_7_ (0.85) (**Supplementary Table S4**). In terms of the b* value, a decreasing trend was noted. T_1_ exhibited the highest b* value (14.87) at the end of the storage period, while the lowest b* value was observed in T_4_ (9.82) for whole garlic bulb. In addition, for garlic paste, T_7_ resulted in highest b* value (12.54), followed by T_10_ (11.83), at the end of the storage period, while the lowest b* value was found in T_4_ (8.69) (**Supplementary Table S5**). At 36 DAST, T_1_ resulted in maximum chroma (14.97), while minimum chroma was found in T_4_ (9.84) (**Supplementary Table S5**). T_15_ exhibited the highest hue angle (97.16), while the lowest hue angle was observed in T_8_ (82.36) (**Supplementary Table S6**). T_1_ demonstrated the highest browning index (15.51), whereas the lowest browning index was observed in T_15_ (6.45) for whole garlic bulb. For garlic paste, at 36 DAST, T_7_ demonstrated maximum chroma (12.57), while the lowest chroma was found in T_4_ (8.97). T_12_ showed the highest hue angle (87.33), while the lowest hue angle was found in T_4_ (75.58). T_11_ gave the highest browning index (11.27), while the lowest browning index was observed in T_12_ (9.27) (**Supplementary Table S8**). The physiological weight loss increased with prolonged storage period. At 36 DAST, T_15_ exhibited the highest percentage of physiological weight loss (4.19), while the lowest percentage of physiological weight loss was observed in T_8_ (1.30) (**Figure 5**).

## 4 Discussion

Seaweed extract had a significant influence on the growth yield, quality, and storage behavior of garlic. It is an essential biostimulant that helps improve the nutrient uptake capacity of the plant and also helps fight against biotic and abiotic stresses. Rayorath et al. (2008) conducted a study on the promotion of vegetative growth in *Arabidopsis thaliana* through the application of *A. nodosum* extract. Their findings suggest that the observed growth enhancement is attributable to compounds such as auxins and gibberellins, which expressed similar activity to cytokinin within the plant. Sundharaiya et al. (2016) conducted an experiment to determine suitable organic inputs for enhancing the growth and yield of multiplier onions. Foliar application of seaweed extract at 2% resulted in notably higher values of plant height, leaf number, leaf length, and leaf breadth at different stages of growth and at harvest, as well as for yield-related traits.

The yield of a plant is directly influenced by the number of leaves it possesses. A greater number of leaves enhance the photosynthetic mechanism in the plant, leading to the increased accumulation of photosynthetic substances and, ultimately, to a higher yield. Elsagan et al. (2020) reported an enhancement in the number of leaves per plant in garlic obtained with the application of *A. nodosum* extract. Greater leaf length facilitates an expanded surface area for photosynthesis, consequently enhancing the crop yield. A similarity in leaf length was observed by Teshale and Tekeste (2020). Vindya et al. (2023) demonstrated an average neck thickness of 5.58 mm when evaluating different genotypes. A similar trend in polar diameter was reported by Shibana and Menon (2019), where six genotypes varied in the range from 27.00 to 34.32 mm. The average equatorial diameter of different germplasm was 33.48 mm. The results are also closely related to the average clove width observed by Tripathi et al. (2021). While evaluating different garlic varieties, a similar range of clove length was found by Prajapati et al. (2016). Sea algae enhanced the number of cloves per bulb, as reported by Elsagan et al. (2020). According to the study by Hidangmayum and Sharma (2017), the highest average fresh weight of onion bulbs was achieved with the application of 0.5% *A. nodosum*.

There was a close correlation in terms of the total yield of the genotypes of garlic (Verma and Thakre (2018). Higher concentrations of seaweed extract significantly enhanced the total yield of garlic (Osman et al., 2021). Similarly, Hidangmayum and Sharma (2017) documented that *A. nodosum* extract resulted in an increment in the bulb yield of onion plants. This enhancement in bulb yield is attributed to the positive effects of seaweed extract on improving the photosynthetic rates and the nutritional status of garlic plants. These improvements contribute to more efficient mineral translocation, robust root system development, and increased chlorophyll content, collectively promoting plant growth and yield. The accumulated GDD values during the different phenophases of garlic showed that the accumulated GDD utilized by garlic in phenophases vary in all the stages (**Figure 4**). The GDD value was lower during the bulb initiation to the maturity stage compared with the vegetative to the bulb initiation stage, which may occur due to the lower maximum and minimum temperatures. Eventually, it resulted in a relatively lower yield compared with the yield during favorable conditions. This might be due to the poor translocation of the photosynthetic substances from sink to source. The bulb growth rate improved in a high temperature condition (Steer, 1980). A higher HUE value signifies the efficient dry matter partitioning to the garlic bulb, as HUE is precisely the result of converted heat energy into dry matter, which is dependent on crop, genetic factors, and temporal variations (Dagar et al., 2017; Solanki et al., 2015).

There was a notable increase in TSS observed in the tropics with *A. nodosum* extract (Ali et al., 2016). *A. nodosum* increased the ascorbic acid content in garlic in comparison to the control (Abbas et al., 2020). Fan et al. (2011) reported that *A. nodosum* extract enhanced the flavonoid synthesis in plant. As a result, it increased the total phenol content in *Spinacia oleracea*. Chan et al. (2014) determined a total phenol content in fresh garlic bulb of 154 ± 10 mg GAE/100 g. Abbas et al. (2020) observed differing responses among four onion cultivars—”Lambada,” “RedBone,” “Nasarpuri,” and “Phulkara”—to varying levels of commercial seaweed extract in terms of their vegetative growth, reproductive behavior, and qualitative attributes. The study revealed that the application of 0.5% seaweed extract resulted in increased yield, improved nutritional content, and higher levels of TSS across all four onion cultivars. Conversely, the maximum concentration examined, i.e., 3% seaweed extract, notably increased the ascorbic acid levels in various onion cultivars. Dubey et al. (2010) observed a notable positive and significant correlation between bulb yield and bulb weight in their study. The clove diameter exhibited a direct positive impact on yield (Singh et al., 2011). Sharma et al. (2016) found that the number of cloves, the weight of the bulb, and the height of the garlic plant had the highest direct effects. Tiwari et al. (2022) identified a significant and positive correlation between the gross yield of garlic bulb and TSS. A significant negative correlation between rainfall and a positive correlation between sunshine hours and the tuber bulking stage of potato were reported by Singh et al. (2022).

Enhancing storage life is an important consideration for those crops that undergo physiological weight loss due to being perishable by nature. In the case of garlic, storage is also a matter of concern. There may be some effects of biostimulants in mitigating the storage life of crops. Physiological weight loss may occur due to the change in the biochemical activities within garlic or to variations in the weather conditions during the storage period. Akan and Güneş (2021) documented a 3.91% physiological weight loss observed at 1 month after storage of garlic bulb. Ulianych et al. (2020) also noted that the highest weight loss in garlic cloves occurred during the first month of the storage period. As cell wall dismantling progresses with maturity, there is a corresponding decrease in the L* value and an increase in the positive a* value (indicating redness) (Domínguez et al., 2011; Liu et al., 2012). A similar trend was observed during the aging of garlic in terms of the L* value (Kang et al., 2022). The hue angle quantifies the proportions of redness and yellowness, with 0°C/360°C indicating red/magenta, 90°C for yellow, 180°C for green, and an angle of 270°C for blue or purple, as well as intermediary hues between these fundamental colors (Kortei and Akonor, 2015). The hue angle is utilized to assess the color quality (Yildiz and Baysal, 2007). The browning index is significant in enzymatic browning processes, requiring the determination of its velocity constants. Hasan et al. (2017) demonstrated that the weight loss percentage of green garlic bulbs increased notably and consistently as the storage period was extended. They observed a significant decrease in the lightness (L*) and hue angle (*h*°C) in the studied treatments with prolonged storage period. The reduction in the L* values might be linked to water loss, which had a negative impact on the brightness of fresh garlic bulbs. In addition, the decrease in the hue angle could be attributed to the emergence of yellowish and reddish tones related to senescence.

## 5 Conclusion

Considering the entire experiment, it can be stated that AVT-1 GNB-23-41 is the superior genotype and that treatment AVT-1 GNB-23-41 × *A. nodosum* at 1 ml/L is the best treatment in terms of yield. In terms of storage, the AVT-1 GNB-23-47 × *A. nodosum* at 1 ml/L treatment is superior, which had the lowest percentage of physiological weight loss at 36 days after storage.

## Data Availability

The original contributions presented in the study are included in the article/**Supplementary Material**. Further inquiries can be directed to the corresponding author.
