# Supplementary material for: The magic of seaweed (Ascophyllum nodosum) extract in influencing the dynamics of yield, quality, and storage behavior of garlic
Source: Front Plant Sci. 2025 Sep 29;16:1636319. doi: 10.3389/fpls.2025.1636319 (PMC12515956; doi:10.3389/fpls.2025.1636319)
Supplement: Supplementary file 1 [file DataSheet1.pdf]

The magic of seaweed (*Ascophyllum nodosum*) extract in influencing the dynamics of yield, quality and storage behavior of garlic

Aniket Mandal[1], Amit Baran Sharangi[1*], Reem Binsuwaidan[2], Safia Obaidur[3], Mohd Saeed[3], Nawaf Alshammari[4], Mamdouh Alshammari[4] and Nadiyah M. Alabdallahd[5,6]

SUPPLEMETARY DOCUMENT

Suppl. Table_1: Physical attributes of the initial soil of experimental field

Distribution of Particle Size	Values
Sand (%)	41.32
Silt (%)	30.01
Clay (%)	28.66
Textural Class	Clay loam
Bulk Density (g/cm^3)	1.57
Paricle Density (g/cm^3)	2.47
Total porosity (%)	40.30
Maximum water holding capacity	37.84

Garlic genotypes

Five different genotypes were collected from National Horticultural Research and Development Foundation, Nasik, Maharashtra and one local variety was taken from Mohanpur market.

Suppl. Table_2: Garlic genotypes and their sources

Name of Genotype	Source
AVT-1 GNB-23-38	NHRDF,Nasik
AVT-1 GNB-23-41	NHRDF,Nasik
AVT-1 GNB-23-47	NHRDF,Nasik
AVT-1 GNB-23-20	NHRDF,Nasik
AVT-1 GNB-23-26	NHRDF,Nasik
Goldana (local variety)	Mohanpur market (Mohanpur, Nadia)

Seaweed (*Ascophyllum nodosum*) extract

Biovita, a commercial seaweed extract obtained from *Ascophyllum nodosum* was used for this experiment. This extract was purchased from a local agro-chemical shop of Kalyani,Nadia, West Bengal.

Suppl. Figure_1: Experimental site

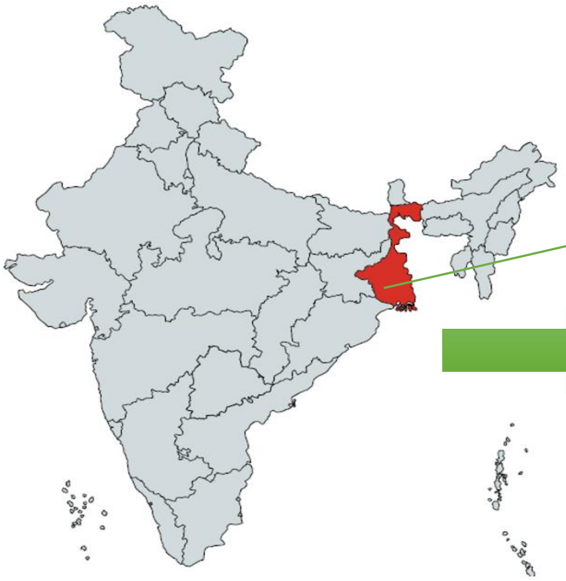

A

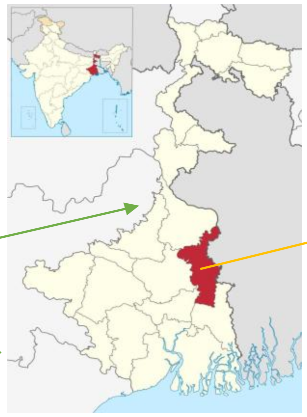

B

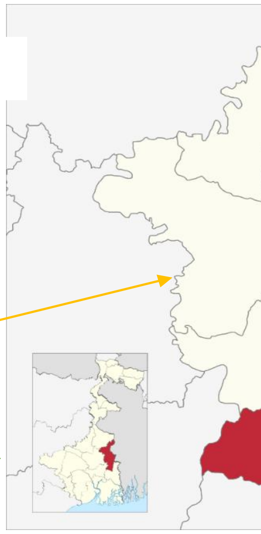

C

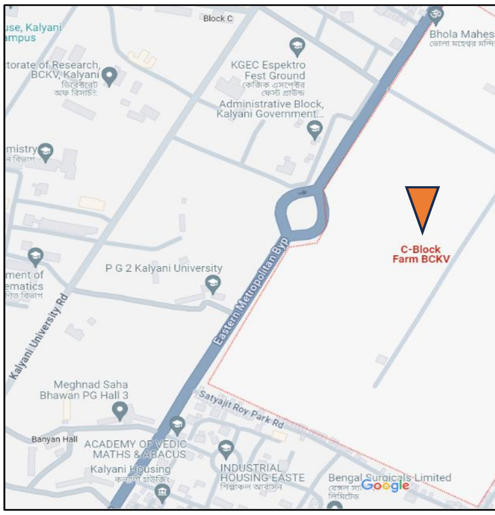

EXPERIMENTAL SITE = ▽

West Bengal within INDIA = A

Nadia district within West Bengal = B

Kalyani subdivision within Nadia district = C

"C" block farm, BCKV within Kalyani subdivision = D

[illegible]

1. **Ascorbic acid (mg /100 g)**

To estimate the ascorbic acid content in garlic, titration against 2,6-dichlorophenol-indophenol dye was conducted following the method described by Ranganna (2001). The results were expressed as mg per 100 g of bulb.

Dye preparation : 150 mL of distilled water was heated in a 200 mL beaker. Then, 42 mg of sodium bicarbonate and 2,6-dichlorophenol-indophenol dye were added. After thorough mixing, the volume was adjusted to 200 mL with distilled water. The solution was stored in an open bottle wrapped with black paper to prevent light penetration and kept in the refrigerator. Standardization was performed before use.

Preparation of 3% metaphosphoric acid: 30 g of metaphosphoric acid was dissolved in a 1 L volumetric flask, and the volume was adjusted to 1000 mL with distilled water.

Standard ascorbic acid solutions were prepared as follows:

- Standard solution A : 100 mL volumetric flask filled with 3% metaphosphoric acid.
- Standard solution B: Another 10 mL solution was placed in a separate 100 mL volumetric flask and volume make up was done with metaphosphoric acid.

Standardization of the dye: Standardization of the dye involved taking freshly prepared dye in a micro burette and adjusting it to "zero(0)". Then, 5 mL of standard B solution was taken in a beaker or conical flask and titrated until a light pink colour appeared.

$$\text{Dye factor} = \frac{0.5}{Average\ \ burette\ \ reading\ \ for\ standardization\ \ \ of\ dye}$$

Preparation of sample : Sample preparation involved taking 10 mL of garlic juice in a 100 mL volumetric flask and diluting it to 100 mL with 3% metaphosphoric acid. Titration against the dye was then conducted until a light pink colour appeared, and the burette reading was recorded.

Calculation:

Ascorbic acid (mg /100 g)

$$= \frac{Burette\ \ reading\ \ \times dye\ factor\ \times volume\ \ made\ \ up}{Volume\ \ of\ sample\ \ taken\ \ for\ estimation\ \ \times weig\,ht\ of\ sample} \times 100$$

2. **Total phenol (mg GAE /100g)**

Reagents:

- Folin & Ciocalteu's phenol reagent
- Gallic acid
- 7.5% Sodium carbonate
- Methanol
- Ethanol

Gallic acid calibration standards:

To prepare a range of gallic acid standards, start by dissolving 0.5 grams of gallic acid in 10 millilitres of ethanol. Following this, dilute the solution with water to a total volume of 100 millilitres, resulting in a final concentration of 5 grams per litre. Subsequently, proceed to dilute 1, 2, 5, and 10 millilitres of this prepared solution individually with 100 millilitres of water to obtain standards with concentrations of 50, 100, 250, and 500 milligrams per litre, respectively.

Process:

The concentration of total phenolics was determined using a modified version of the method outlined by Singleton and Rossi (1965). Initially, 1 ml of aliquot of appropriately diluted extracts or different standard solutions of gallic acid (0, 50, 100, 250, 500 mg/L) was introduced into a 25 ml volumetric flask which contained 9 ml of ddH_2O. A reagent blank was also prepared using ddH_2O. Following this, 1 ml of Folin & Ciocalteu's phenol reagent was added to the mixture and thoroughly mixed. After a 5-minute incubation period, 10 ml of 7% Na_2CO_3 solution was added with continuous mixing. The solution was promptly diluted to a final volume of 25 ml with ddH_2O and mixed thoroughly once again. Subsequently, the mixture was allowed to incubate for 90 minutes, and then the absorbance was measured at 750 nm against the prepared blank.

Calculation:

The concentration of TPC was expressed as GAEs by reference to the GA standard calibration curve. The calibration equation for Gallic acid was $y= 0.0056x + 0.0275$, ($R^2 = 0.9999$), where x is the GA concentration in mg/l and y is the absorbance reading at 750nm

The total phenolic content (TPC) was quantified in terms of Gallic Acid Equivalents (GAEs) using the Gallic acid standard calibration curve. The calibration equation for Gallic acid was determined as y = 0.005x + 0.027,(R^2 = 0.9999), where 'x' represents the concentration of Gallic acid in milligrams per litre (mg/L), and 'y' denotes the absorbance reading at 750nm. (**Suppl. Figure_2**).

The total phenolic content of all the samples was computed the using the formula:

$$C = c\,\frac{V}{m}$$

where, "C" represents the total phenolic content in milligrams per 100 grams in terms of Gallic Acid Equivalent (GAE). "c" denotes the concentration of gallic acid derived from the calibration curve, expressed in milligrams per millilitre (mg/ml). "v" signifies the volume of the extract in millilitres, while "m" represents the mass of the extract in grams.

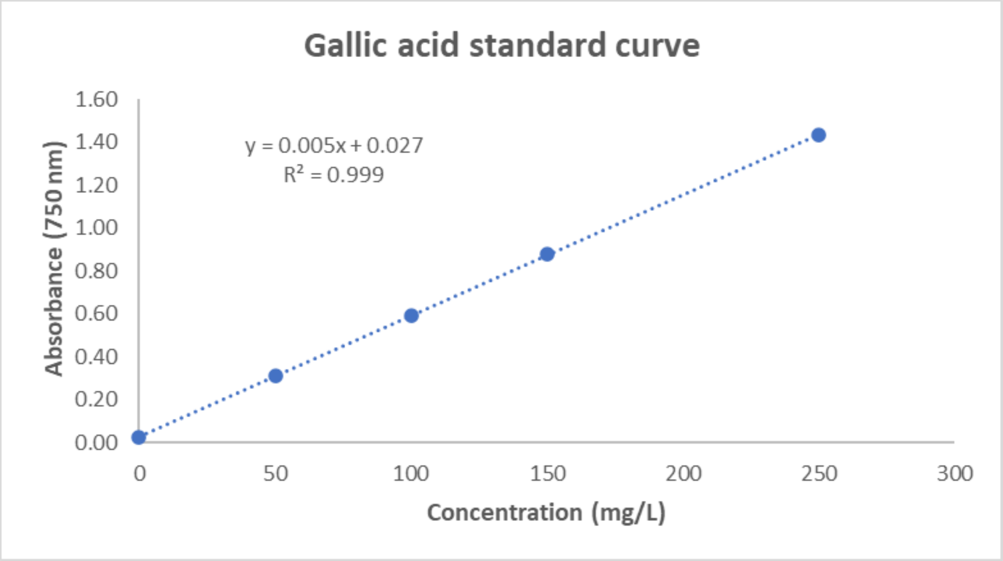

Suppl. Figure_2 : Gallic acid standard curve

Colour/Chromaticity values

3.4.5.2.6.1 L*, a*, b* values

The L*, a*, b* colour values were obtained using a Hunter colorimeter, specifically the Colour Quest XE, which functions as a dual-beam xenon flash spectrophotometer with a spectral range spanning from 400 to 700 nanometres (nm). This instrument facilitates measurements of either

the reflected or transmitted colour of a given product. Its sensor employs a 152.4 mm diameter plastic integrating sphere coated with Spectraflect™, ensuring uniform diffusion of light emitted by the lamp. The light interacts with the sample, either reflecting from its surface or transmitting through it. Prior to measurements, the colorimeter was calibrated using the manufacturer's standard white calibrated tile to ensure accurate colour perception. The colour changes were quantified within the L*, a*, b* colour space, a widely utilized colour model. In this model, the L* value represents the luminance or brightness, with higher values indicating greater brightness and lower values indicating darkness. The a* value denotes the position on the red-green axis, with positive values indicating a tendency towards red-purple hues and negative values indicating a tendency towards green. Conversely, the b* value signifies the position on the yellow-blue axis, where positive values suggest a leaning towards yellow and negative values indicate a tendency towards blue. The experimental setup utilized whole garlic bulbs and garlic paste as sample materials for colour assessment.

3. Chroma, Hue Angle and Browning index (BI)

3.1 Chroma

Chroma, also referred to as saturation, serves as a quantitative metric for evaluating the intensity or vividness of a colour. It indicates the degree to which a colour appears pure or vivid, and is directly correlated with the colour's strength or richness.

$$C = \sqrt{a^2 + b^2}$$

3.2 Hue Angle (°)

The Hue angle serves as a common parameter for characterizing the colour of food products. Over the course of the storage period, changes in the hue angle value indicate shifts in colour perception, often associated with hues such as red, orange, yellow, green, blue, or violet. Notably, a hue angle between 0° and 360° typically corresponds to a red hue, while angles of 90°, 180°, and 270° represent yellow, green, and blue hues, respectively.

$$H = \tan^{-1}(\frac{b}{a})$$

3.3 Browning index (BI)

The Browning index is an essential parameter linked to the intensity of brown coloration and is widely regarded as a crucial metric in browning assessments (Ding *et al.*, 2020).

$$\text{BI} = \frac{[100(X-0.31)]}{0.17}$$

$$\text{Where, x} = \frac{(a+1.75L)}{5.645L+a-0.3012b}$$

Suppl. Table_3: L* value of whole garlic bulb and garlic paste at different storage interval

**Treatments**	**L* value**													
	**0 DAST**		**6 DAST**		**12 DAST**		**18 DAST**		**24 DAST**		**30 DAST**		**36 DAST**	
	WGB	GP	WGB	GP	WGB	GP	WGB	GP	WGB	GP	WGB	GP	WGB	GP
T_1	64.43	62.82	55.02	60.34	49.54	58.62	37.61	56.41	34.48	53.67	32.32	51.82	30.29	47.50
T_2	67.92	63.36	56.84	61.23	48.56	59.28	35.56	58.28	33.24	57.24	31.71	55.00	29.47	50.43
T_3	72.91	61.24	65.24	59.43	59.49	57.73	49.24	56.97	42.05	55.08	38.17	53.02	35.14	48.03
T_4	63.30	63.76	62.77	60.32	51.50	58.53	43.51	58.34	38.15	56.15	36.05	54.29	34.38	52.09
T_5	59.54	64.26	58.41	62.90	54.09	60.84	44.27	59.75	40.15	58.43	38.13	56.00	36.54	52.26
T_6	60.69	60.19	54.24	59.84	41.57	58.23	34.07	57.15	32.63	55.45	31.11	54.26	30.14	51.31
T_7	62.51	67.12	60.73	64.16	51.47	62.65	43.64	59.73	39.23	56.19	37.83	53.73	37.09	52.34
T_8	69.46	63.68	67.36	61.57	52.89	60.06	45.59	59.23	40.52	58.05	39.06	56.56	36.79	52.73
T_9	66.77	59.51	61.54	57.30	50.92	55.72	44.76	50.67	39.66	56.26	37.48	54.70	35.27	49.62
T_{10}	58.64	58.71	57.31	57.30	41.71	56.58	36.64	58.12	33.04	56.63	32.06	55.33	29.82	52.73
T_{11}	66.45	62.45	65.58	60.36	52.36	58.82	46.38	57.11	41.72	56.00	39.61	54.70	38.42	49.82
T_{12}	61.39	62.16	58.55	60.16	48.74	57.83	38.94	55.78	35.81	54.49	33.63	53.21	31.54	50.60
T_{13}	68.68	60.86	67.05	59.19	57.00	58.89	46.62	57.73	36.39	55.48	32.68	52.89	30.79	49.33
T_{14}	71.90	67.29	69.07	65.09	57.85	62.18	48.70	61.81	41.31	59.08	38.15	52.14	36.70	50.01
T_{15}	67.27	64.22	65.83	62.16	59.41	60.56	48.05	59.81	42.04	57.30	38.64	50.46	36.49	51.66
T_{16}	60.53	59.14	58.34	57.56	49.22	56.63	41.25	54.85	36.47	52.68	35.14	51.69	33.39	49.84
**MEAN**	65.15	62.55	61.49	60.56	51.65	58.95	42.80	57.61	37.93	56.14	35.74	53.74	33.89	50.64
**S.Em(±)**	0.180	0.082	0.060	0.042	0.204	0.056	0.118	0.049	0.072	0.108	0.103	0.104	0.156	0.284
**$CD_{0.05}$**	0.524	0.239	0.183	0.123	0.591	0.163	0.341	0.142	0.209	0.314	0.298	0.302	0.454	0.824

Here, WGB = Whole garlic bulb, GP= Garlic paste, DAST = Days after storage, T_1 = AVT-1 GNB-23-38+ *A. nodosum* @ 0.5 ml/L, T_2 = AVT-1 GNB-23-38+ *A. nodosum* @ 1 ml/L,T_3 = AVT-1 GNB-23-38+ *A. nodosum* @ 1.5 ml/L, T_4 = AVT-1 GNB-23-41+ *A. nodosum* @ 0.5 ml/L , T_5 = AVT-1 GNB-23-41+ *A. nodosum* @ 1 ml/L,T_6 = AVT-1 GNB-23-41+ *A. nodosum* @ 1.5 ml/L, T_7 = AVT-1 GNB-23-47+ *A. nodosum* @ 0.5 ml/L , T_8 = AVT-1 GNB-23-47+ *A. nodosum* @ 1 ml/L,T_9 = AVT-1 GNB-23-47+ *A. nodosum* @ 1.5 ml/L, T_{10} = AVT-1 GNB-23-20+ *A. nodosum* @ 0.5 ml/L , T_{11} = AVT-1 GNB-23-20+ *A. nodosum* @ 1 ml/L,T_{12} = AVT-1 GNB-23-20+ *A. nodosum* @ 1.5 ml/L, T_{13} = AVT-1 GNB-23-26+ *A.*

nodosum @ 0.5 ml/L , T_{14} = AVT-1 GNB-23-26+ *A. nodosum* @ 1 ml/L,T_{15} = AVT-1 GNB-23-26+ *A. nodosum* @ 1.5 ml/L, T_{16} = Goldana (local variety)+ no spray.

Suppl. Table_4: a* value of whole garlic bulb and garlic paste at different storage interval

Treatments	a* value													
	0 DAST		6 DAST		12 DAST		18 DAST		24 DAST		30 DAST		36 DAST	
	WGB	GP	WGB	GP	WGB	GP	WGB	GP	WGB	GP	WGB	GP	WGB	GP
T_1	-0.63	-3.58	0.18	-3.14	3.51	-1.40	2.91	-0.74	2.14	1.71	1.86	1.53	1.76	1.26
T_2	-1.40	-3.55	-1.34	-2.67	3.98	-1.53	3.23	1.02	2.75	2.11	2.24	1.89	0.57	1.56
T_3	-1.68	-3.85	-0.57	-3.29	2.56	-2.54	1.94	-1.14	1.23	2.60	0.54	2.24	0.36	2.08
T_4	-2.01	-2.99	-2.03	-1.73	2.85	-0.64	2.16	1.05	1.52	2.55	0.66	2.29	-0.58	2.23
T_5	-1.16	-3.53	-1.03	-3.59	4.53	-2.07	3.47	-0.65	2.37	1.54	1.96	1.33	1.14	1.09
T_6	-1.33	-3.64	-1.17	-2.85	2.84	-1.15	2.16	0.57	1.86	1.75	1.73	1.29	-0.93	1.13
T_7	-2.42	-3.21	-2.23	-3.32	4.32	-1.96	3.41	-0.07	2.65	1.25	2.14	1.06	0.93	0.85
T_8	-1.82	-2.69	-1.70	-2.05	3.95	-0.38	3.15	1.09	2.45	3.14	2.16	2.62	1.89	1.92
T_9	-1.63	-3.56	-1.53	-3.04	2.47	-2.50	1.82	0.88	1.33	2.05	-0.15	1.62	-0.74	1.29
T_{10}	-1.25	-3.76	-1.02	-3.17	3.15	-2.00	2.25	0.98	1.62	2.74	1.72	2.48	1.42	1.64
T_{11}	-1.59	-3.02	-1.44	-2.43	3.02	-0.18	2.11	1.04	1.57	2.97	1.46	2.54	-0.57	1.98
T_{12}	-2.14	-4.09	-2.14	-3.17	2.16	-2.65	1.01	-0.92	-0.14	1.21	0.25	0.87	-0.48	0.55
T_{13}	-1.92	-2.86	-1.45	-2.44	2.56	-1.50	1.46	0.46	0.83	1.26	0.86	1.02	-0.86	0.90
T_{14}	-0.43	-3.27	-0.40	-1.60	2.35	-0.43	1.38	0.46	0.76	1.36	0.83	1.14	0.08	0.96

T_{15}	-1.93	-4.10	-1.88	-0.75	2.16	1.06	1.12	1.34	-0.14	2.65	-0.55	2.10	-1.67	1.51
T_{16}	-1.37	-3.49	-1.06	-2.35	3.72	-1.55	2.53	0.08	1.84	1.96	1.60	1.61	1.40	1.26
**MEAN**	-1.54	-3.45	-1.30	-2.60	3.13	-1.34	2.26	0.34	1.54	2.05	1.21	1.73	0.23	1.39
**S.Em(±)**	0.074	0.112	0.070	0.114	0.061	0.059	0.069	0.093	0.053	0.086	0.076	0.054	0.131	0.055
**$CD_{0.05}$**	0.215	0.325	0.204	0.330	0.178	0.171	0.200	0.270	0.153	0.249	0.221	0.156	0.380	0.160

Here, WGB = Whole garlic bulb, GP= Garlic paste, DAST = Days after storage, T_1 = AVT-1 GNB-23-38+ *A. nodosum* @ 0.5 ml/L, T_2 = AVT-1 GNB-23-38+ *A. nodosum* @ 1 ml/L,T_3 = AVT-1 GNB-23-38+ *A. nodosum* @ 1.5 ml/L, T_4 = AVT-1 GNB-23-41+ *A. nodosum* @ 0.5 ml/L , T_5 = AVT-1 GNB-23-41+ *A. nodosum* @ 1 ml/L,T_6 = AVT-1 GNB-23-41+ *A. nodosum* @ 1.5 ml/L, T_7 = AVT-1 GNB-23-47+ *A. nodosum* @ 0.5 ml/L , T_8 = AVT-1 GNB-23-47+ *A. nodosum* @ 1 ml/L,T_9 = AVT-1 GNB-23-47+ *A. nodosum* @ 1.5 ml/L, T_{10} = AVT-1 GNB-23-20+ *A. nodosum* @ 0.5 ml/L , T_{11} = AVT-1 GNB-23-20+ *A. nodosum* @ 1 ml/L,T_{12} = AVT-1 GNB-23-20+ *A. nodosum* @ 1.5 ml/L, T_{13} = AVT-1 GNB-23-26+ *A. nodosum* @ 0.5 ml/L , T_{14} = AVT-1 GNB-23-26+ *A. nodosum* @ 1 ml/L,T_{15} = AVT-1 GNB-23-26+ *A. nodosum* @ 1.5 ml/L, T_{16} = Goldana (local variety)+ no spray.

Suppl. Table_5: b* value of whole garlic bulb and garlic paste at different storage interval

Treatments	b* value													
	0 DAST		6 DAST		12 DAST		18 DAST		24 DAST		30 DAST		36 DAST	
	WGB	GP	WGB	GP	WGB	GP	WGB	GP	WGB	GP	WGB	GP	WGB	GP
T_1	25.11	20.72	21.95	20.20	19.93	18.53	18.63	16.53	17.66	15.38	15.83	13.49	14.87	11.39
T_2	26.22	21.84	22.42	21.23	19.70	18.47	17.08	15.71	15.04	15.30	13.35	14.10	12.17	10.63
T_3	27.79	21.15	25.33	20.11	19.98	18.04	18.16	15.58	16.73	14.75	14.78	11.52	11.25	9.42
T_4	24.66	21.92	24.50	21.59	17.56	19.12	17.22	14.64	14.70	12.36	12.14	10.70	9.82	8.69
T_5	23.79	22.19	23.09	22.09	21.21	20.24	17.81	19.08	15.63	17.00	13.97	14.36	12.44	10.91
T_6	23.88	21.90	21.54	21.58	20.53	19.22	17.84	18.09	16.16	14.83	14.15	13.80	13.16	10.75
T_7	24.45	21.92	23.76	21.34	17.56	20.36	19.34	19.27	18.75	17.79	16.26	15.77	14.41	12.54
T_8	26.33	22.48	25.99	22.03	23.69	20.18	19.13	18.35	18.07	15.83	15.89	13.73	14.12	10.85
T_9	25.90	21.65	24.04	20.89	20.29	17.49	18.01	15.02	17.20	14.46	16.01	13.24	14.12	10.69
T_{10}	23.15	22.02	22.67	21.37	20.38	18.73	19.66	14.58	17.25	14.34	15.13	13.68	12.05	11.83
T_{11}	25.41	21.83	25.34	20.86	20.78	18.03	18.63	16.17	16.93	15.20	13.77	14.17	12.20	11.12

T_{12}	24.44	21.19	23.06	21.05	22.62	19.89	15.67	16.39	13.74	15.63	12.01	13.00	10.19	11.81
T_{13}	26.05	21.15	25.91	19.77	21.84	17.77	18.35	15.59	17.62	14.42	16.19	13.68	14.39	10.37
T_{14}	26.63	21.16	26.56	20.49	22.38	20.21	17.03	18.65	15.87	16.02	13.15	13.97	12.12	11.03
T_{15}	25.58	21.20	25.50	20.54	22.69	19.62	18.74	17.86	17.10	15.35	15.25	13.64	13.29	10.30
T_{16}	26.47	21.33	26.38	20.62	22.57	17.91	18.05	16.28	16.61	14.90	14.65	13.72	13.41	10.42
**MEAN**	25.37	21.60	24.25	20.98	20.86	18.99	18.08	16.74	16.57	15.22	14.53	13.54	12.75	10.80
**S.Em(±)**	0.009	0.033	0.034	0.050	0.032	0.030	0.111	0.157	0.090	0.092	0.194	0.190	0.085	0.150
**$CD_{0.05}$**	0.026	0.094	0.098	0.144	0.092	0.086	0.321	0.456	0.261	0.268	0.564	0.552	0.247	0.435

Here, WGB = Whole garlic bulb, GP= Garlic paste, DAST = Days after storage, T_1 = AVT-1 GNB-23-38+ *A. nodosum* @ 0.5 ml/L, T_2 = AVT-1 GNB-23-38+ *A. nodosum* @ 1 ml/L,T_3 = AVT-1 GNB-23-38+ *A. nodosum* @ 1.5 ml/L, T_4 = AVT-1 GNB-23-41+ *A. nodosum* @ 0.5 ml/L , T_5 = AVT-1 GNB-23-41+ *A. nodosum* @ 1 ml/L,T_6 = AVT-1 GNB-23-41+ *A. nodosum* @ 1.5 ml/L, T_7 = AVT-1 GNB-23-47+ *A. nodosum* @ 0.5 ml/L , T_8 = AVT-1 GNB-23-47+ *A. nodosum* @ 1 ml/L,T_9 = AVT-1 GNB-23-47+ *A. nodosum* @ 1.5 ml/L, T_{10} = AVT-1 GNB-23-20+ *A. nodosum* @ 0.5 ml/L , T_{11} = AVT-1 GNB-23-20+ *A. nodosum* @ 1 ml/L,T_{12} = AVT-1 GNB-23-20+ *A. nodosum* @ 1.5 ml/L, T_{13} = AVT-1 GNB-23-26+ *A. nodosum* @ 0.5 ml/L , T_{14} = AVT-1 GNB-23-26+ *A. nodosum* @ 1 ml/L,T_{15} = AVT-1 GNB-23-26+ *A. nodosum* @ 1.5 ml/L, T_{16} = Goldana (local variety)+ no spray.

Suppl. Table_6: Chroma of whole garlic bulb and garlic paste at different storage interval

**Treatments**	**Chroma**													
	**0 DAST**		**6 DAST**		**12 DAST**		**18 DAST**		**24 DAST**		**30 DAST**		**36 DAST**	
	WGB	GP	WGB	GP	WGB	GP	WGB	GP	WGB	GP	WGB	GP	WGB	GP
T_1	25.11	21.03	21.95	20.44	20.24	18.58	18.86	16.55	17.79	15.47	15.94	13.58	14.97	11.46
T_2	26.26	22.13	22.46	21.40	20.10	18.53	17.38	15.74	15.28	15.45	13.53	14.23	12.18	10.75
T_3	27.84	21.49	25.33	20.38	20.15	18.21	18.26	15.63	16.78	14.97	14.79	11.74	11.26	9.65
T_4	24.75	22.12	24.59	21.66	17.79	19.13	17.35	14.68	14.78	12.62	12.16	10.94	9.84	8.97
T_5	23.81	22.47	23.11	22.38	21.69	20.34	18.14	19.09	15.81	17.07	14.10	14.42	12.49	10.97
T_6	23.92	22.20	21.57	21.77	20.73	19.25	17.97	18.10	16.26	14.93	14.25	13.86	13.19	10.81
T_7	24.57	22.16	23.86	21.60	18.09	20.45	19.64	19.27	18.94	17.83	16.40	15.81	14.44	12.57
T_8	26.40	22.64	26.04	22.12	24.02	20.18	19.39	18.38	18.24	16.14	16.03	13.98	14.24	11.02
T_9	25.94	21.94	24.10	21.11	20.44	17.67	18.10	15.05	17.26	14.60	16.01	13.34	14.14	10.77

T_{10}	23.19	22.34	22.70	21.60	20.62	18.83	19.78	14.62	17.33	14.60	15.23	13.91	12.14	11.94
T_{11}	25.46	22.03	25.38	21.00	21.00	18.03	18.75	16.21	17.00	15.48	13.85	14.40	12.22	11.30
T_{12}	24.54	21.57	23.15	21.29	22.73	20.07	15.70	16.42	13.74	15.67	12.01	13.03	10.21	11.82
T_{13}	26.12	21.35	25.95	19.92	21.99	17.83	18.41	15.60	17.64	14.47	16.21	13.72	14.41	10.41
T_{14}	26.63	21.41	26.57	20.55	22.50	20.21	17.08	18.66	15.89	16.08	13.17	14.01	12.12	11.08
T_{15}	25.65	21.59	25.57	20.55	22.79	19.65	18.78	17.91	17.10	15.58	15.26	13.81	13.40	10.41
T_{16}	26.51	21.61	26.40	20.75	22.87	17.98	18.22	16.28	16.71	15.03	14.74	13.81	13.49	10.50
**MEAN**	25.42	21.88	24.30	21.16	21.11	19.06	18.24	16.76	16.66	15.38	14.60	13.66	12.80	10.90
**S.Em(±)**	0.014	0.038	0.030	0.050	0.031	0.032	0.108	0.156	0.088	0.091	0.195	0.186	0.084	0.148
**$CD_{0.05}$**	0.042	0.111	0.088	0.146	0.089	0.092	0.314	0.453	0.256	0.265	0.565	0.541	0.244	0.429

Here, WGB = Whole garlic bulb, GP= Garlic paste, DAST = Days after storage, T_1 = AVT-1 GNB-23-38+ *A. nodosum* @ 0.5 ml/L, T_2 = AVT-1 GNB-23-38+ *A. nodosum* @ 1 ml/L,T_3 = AVT-1 GNB-23-38+ *A. nodosum* @ 1.5 ml/L, T_4 = AVT-1 GNB-23-41+ *A. nodosum* @ 0.5 ml/L , T_5 = AVT-1 GNB-23-41+ *A. nodosum* @ 1 ml/L,T_6 = AVT-1 GNB-23-41+ *A. nodosum* @ 1.5 ml/L, T_7 = AVT-1 GNB-23-47+ *A. nodosum* @ 0.5 ml/L , T_8 = AVT-1 GNB-23-47+ *A. nodosum* @ 1 ml/L,T_9 = AVT-1 GNB-23-47+ *A. nodosum* @ 1.5 ml/L, T_{10} = AVT-1 GNB-23-20+ *A. nodosum* @ 0.5 ml/L , T_{11} = AVT-1 GNB-23-20+ *A. nodosum* @ 1 ml/L,T_{12} = AVT-1 GNB-23-20+ *A. nodosum* @ 1.5 ml/L, T_{13} = AVT-1 GNB-23-26+ *A. nodosum* @ 0.5 ml/L , T_{14} = AVT-1 GNB-23-26+ *A. nodosum* @ 1 ml/L,T_{15} = AVT-1 GNB-23-26+ *A. nodosum* @ 1.5 ml/L, T_{16} = Goldana (local variety)+ no spray.

Suppl. Table_7: Hue angle (°) of whole garlic bulb and garlic paste at different storage interval

**Treatments**	**Hue angle (°)**													
	**0 DAST**		**6 DAST**		**12 DAST**		**18 DAST**		**24 DAST**		**30 DAST**		**36 DAST**	
	WGB	GP	WGB	GP	WGB	GP	WGB	GP	WGB	GP	WGB	GP	WGB	GP
T_1	91.43	99.79	89.52	98.84	80.00	94.30	81.12	92.56	83.09	83.64	83.29	83.54	83.26	83.66
T_2	93.05	99.23	93.43	97.16	78.58	94.72	79.28	86.30	79.64	82.14	80.47	82.37	87.31	81.65
T_3	93.46	100.32	91.29	99.29	82.70	98.01	83.91	94.17	85.78	80.00	87.92	79.00	88.14	77.55
T_4	94.66	97.76	94.74	94.58	80.78	91.91	82.85	85.92	84.10	78.32	86.89	77.90	93.38	75.58
T_5	92.79	99.03	92.56	99.21	77.94	95.82	78.99	91.96	81.39	84.83	82.01	84.71	84.77	84.28
T_6	93.19	99.42	93.09	97.52	82.13	93.41	83.10	88.18	83.44	83.27	83.01	84.66	94.03	84.00
T_7	95.6	98.3	95.3	98.	76.1	95.	80.0	90.	81.9	85.	82.4	86.	86.3	86.

	5	3	5	83	8	49	0	20	7	98	9	15	0	13
T_8	93.96	96.81	93.74	95.32	80.52	91.07	80.64	86.59	82.27	78.79	82.27	79.19	82.36	79.95
T_9	93.60	99.33	93.64	98.26	83.06	98.12	84.22	86.65	85.57	81.94	90.53	83.01	92.98	83.10
T_{10}	93.08	99.70	92.58	98.43	81.22	96.09	83.46	86.15	84.64	79.19	83.50	79.72	83.31	82.08
T_{11}	93.59	97.87	93.24	96.65	81.74	90.58	83.53	86.32	84.71	78.94	83.95	79.78	92.65	79.89
T_{12}	95.00	100.91	95.29	98.56	84.55	97.59	86.30	93.20	90.58	85.56	88.79	86.17	92.72	87.33
T_{13}	94.21	97.70	93.20	97.03	83.31	94.81	85.45	88.29	87.29	85.01	86.95	85.74	93.42	85.02
T_{14}	90.92	98.79	90.86	94.46	84.01	91.21	85.38	88.58	87.26	85.16	86.41	85.34	89.61	85.03
T_{15}	94.31	100.93	94.22	92.08	84.56	86.92	86.57	85.71	90.46	80.19	92.07	81.26	97.16	81.68
T_{16}	92.96	99.30	92.30	96.50	80.65	94.93	82.01	89.73	83.67	82.52	83.77	83.32	84.03	83.12
**MEAN**	93.49	99.08	93.07	97.05	81.37	94.06	82.93	88.78	84.74	82.22	85.27	82.62	89.09	82.50
**S.Em(±)**	0.172	0.291	0.168	0.307	0.172	0.179	0.231	0.335	0.194	0.339	0.284	0.276	0.620	0.323
**$CD_{0.05}$**	0.499	0.845	0.488	0.890	0.500	0.518	0.670	0.973	0.564	0.982	0.824	0.801	1.799	0.938

Here, WGB = Whole garlic bulb, GP= Garlic paste, DAST = Days after storage, T_1 = AVT-1 GNB-23-38+ *A. nodosum* @ 0.5 ml/L, T_2 = AVT-1 GNB-23-38+ *A. nodosum* @ 1 ml/L,T_3 = AVT-1 GNB-23-38+ *A. nodosum* @ 1.5 ml/L, T_4 = AVT-1 GNB-23-41+ *A. nodosum* @ 0.5 ml/L , T_5 = AVT-1 GNB-23-41+ *A. nodosum* @ 1 ml/L,T_6 = AVT-1 GNB-23-41+ *A. nodosum* @ 1.5 ml/L, T_7 = AVT-1 GNB-23-47+ *A. nodosum* @ 0.5 ml/L , T_8 = AVT-1 GNB-23-47+ *A. nodosum* @ 1 ml/L,T_9 = AVT-1 GNB-23-47+ *A. nodosum* @ 1.5 ml/L, T_{10} = AVT-1 GNB-23-20+ *A. nodosum* @ 0.5 ml/L , T_{11} = AVT-1 GNB-23-20+ *A. nodosum* @ 1 ml/L,T_{12} = AVT-1 GNB-23-20+ *A. nodosum* @ 1.5 ml/L, T_{13} = AVT-1 GNB-23-26+ *A. nodosum* @ 0.5 ml/L , T_{14} = AVT-1 GNB-23-26+ *A. nodosum* @ 1 ml/L,T_{15} = AVT-1 GNB-23-26+ *A. nodosum* @ 1.5 ml/L, T_{16} = Goldana (local variety)+ no spray.

Suppl. Table_8: Browning index of whole garlic bulb and garlic paste at different storage interval

**Treatments**	**Browning index**													
	**0 DAST**		**6 DAST**		**12 DAST**		**18 DAST**		**24 DAST**		**30 DAST**		**36 DAST**	
	WGB	GP	WGB	GP	WGB	GP	WGB	GP	WGB	GP	WGB	GP	WGB	GP
T_1	9.43	5.25	10.49	5.67	15.47	7.59	16.96	8.13	16.04	11.38	15.47	10.91	15.51	10.49
T_2	8.58	5.47	8.44	6.43	16.30	7.40	17.76	10.13	16.86	11.53	15.65	11.22	11.82	10.49
T_3	8.35	4.97	9.48	5.46	12.72	6.04	12.81	7.42	12.40	12.28	11.15	11.38	10.16	11.23
T_4	7.78	6.16	7.74	7.67	13.67	8.66	13.85	9.97	13.03	11.64	10.92	11.16	7.78	10.89
T_5	8.81	5.5	8.90	5.4	16.2	7.0	16.0	8.5	14.4	11.	13.6	10.	11.9	9.7

		7		7	8	0	0	8	6	02	5	46	0	4
T_6	8.56	5.35	8.63	6.28	16.28	8.05	16.26	10.09	15.47	11.14	14.94	10.43	8.36	9.83
T_7	7.28	5.90	7.43	5.67	15.73	7.13	16.44	9.34	16.05	10.99	14.74	10.56	11.99	9.72
T_8	8.08	6.61	8.23	7.32	16.24	9.10	15.53	10.64	15.19	12.83	14.38	11.95	13.85	10.84
T_9	8.31	5.39	8.31	5.92	13.80	5.99	13.27	10.41	13.10	11.39	10.26	10.73	8.71	10.19
T_{10}	8.63	5.19	8.89	5.84	16.74	6.89	16.27	9.89	15.20	12.21	14.99	11.89	13.79	10.65
T_{11}	8.28	6.10	8.48	6.67	14.44	9.02	13.61	10.34	13.08	12.74	12.40	12.13	8.28	11.27
T_{12}	7.64	4.70	7.47	5.79	14.21	6.24	12.20	7.89	9.79	10.67	10.35	9.78	8.30	9.27
T_{13}	7.95	6.19	8.51	6.47	13.37	7.31	12.49	9.45	12.87	10.41	13.25	10.15	8.92	9.56
T_{14}	9.50	5.71	9.66	7.52	13.09	8.90	11.79	9.75	11.44	10.56	11.26	10.44	9.67	9.74
T_{15}	7.91	4.73	7.99	8.62	12.74	10.72	11.87	10.81	10.09	12.22	9.14	11.90	6.45	10.24
T_{16}	8.98	5.42	9.47	6.77	16.41	7.33	15.17	9.25	14.57	11.72	13.79	11.09	13.36	10.06
**MEAN**	8.38	5.54	8.63	6.47	14.84	7.71	14.52	9.51	13.73	11.55	12.90	11.01	10.55	10.26
**S.Em(±)**	0.089	0.137	0.091	0.142	0.100	0.075	0.110	0.115	0.100	0.111	0.177	0.081	0.289	0.084
**$CD_{0.05}$**	0.258	0.398	0.263	0.411	0.289	0.219	0.320	0.334	0.291	0.322	0.515	0.236	0.839	0.243

Here, WGB = Whole garlic bulb, GP= Garlic paste, DAST = Days after storage, T_1 = AVT-1 GNB-23-38+ *A. nodosum* @ 0.5 ml/L, T_2 = AVT-1 GNB-23-38+ *A. nodosum* @ 1 ml/L,T_3 = AVT-1 GNB-23-38+ *A. nodosum* @ 1.5 ml/L, T_4 = AVT-1 GNB-23-41+ *A. nodosum* @ 0.5 ml/L , T_5 = AVT-1 GNB-23-41+ *A. nodosum* @ 1 ml/L,T_6 = AVT-1 GNB-23-41+ *A. nodosum* @ 1.5 ml/L, T_7 = AVT-1 GNB-23-47+ *A. nodosum* @ 0.5 ml/L , T_8 = AVT-1 GNB-23-47+ *A. nodosum* @ 1 ml/L,T_9 = AVT-1 GNB-23-47+ *A. nodosum* @ 1.5 ml/L, T_{10} = AVT-1 GNB-23-20+ *A. nodosum* @ 0.5 ml/L , T_{11} = AVT-1 GNB-23-20+ *A. nodosum* @ 1 ml/L,T_{12} = AVT-1 GNB-23-20+ *A. nodosum* @ 1.5 ml/L, T_{13} = AVT-1 GNB-23-26+ *A. nodosum* @ 0.5 ml/L , T_{14} = AVT-1 GNB-23-26+ *A. nodosum* @ 1 ml/L,T_{15} = AVT-1 GNB-23-26+ *A. nodosum* @ 1.5 ml/L, T_{16} = Goldana (local variety)+ no spray.

Suppl. Table_9: Morphological characteristics of garlic as influenced by genotype and different doses of *A. nodosum*

Genotype	Morphological characteristics						
	PHH(cm)	NLPPH	LL(cm)	LW(cm)	NT(mm)	PD(mm)	ED(mm)
AVT-1 GNB-23-38	67.57	6.53	40.83	2.08	6.86	27.81	32.32
AVT-1 GNB-23-41	70.92	7.07	44.07	2.11	6.77	34.32	35.12
AVT-1 GNB-23-47	69.63	6.38	44.76	1.99	7.00	27.00	30.13
AVT-1 GNB-23-20	69.50	6.74	41.98	2.00	7.30	30.65	32.43
AVT-1 GNB-23-26	71.14	5.96	43.55	1.79	6.74	33.11	34.29
**Mean**	69.75	6.54	43.04	1.99	6.94	30.58	32.86
**S.Em(±)**	0.41	0.13	0.23	0.01	0.03	0.03	0.02
**$CD_{0.05}$**	1.20	0.38	0.67	0.03	0.07	0.08	0.04
***A. nodosum* dose**							

0.5 ml/L	70.24	6.76	43.43	1.98	6.98	31.66	33.18
1 ml /L	71.00	6.49	43.17	2.11	7.41	30.85	33.50
1.5 ml/L	68.01	6.36	42.51	1.9	6.41	29.23	31.90
**Mean**	69.75	6.54	43.04	2.00	6.94	30.58	32.86
**S.Em(±)**	0.32	0.10	0.18	0.01	0.02	0.02	0.01
**$CD_{0.05}$**	0.93	0.29	0.52	0.02	0.06	0.07	0.03

PHH: Pl ht at harvest; NLPPH: Number of leaves per plant at harvest; LL:Leaf length; LW:Leaf width; NT: Neck thickness; PD: Polar diameter; ED: Equatorial diameter; Values with same letter(s) within a column are not significantly different at P= 0.05 (Duncun's Multiple Range Test, DMRT) . NS = Not Significant at P=0.05.

Suppl. Table_10: Yield and Yield attributing characteristics of garlic as influenced by genotype and different doses of *A. nodosum*

Genotype	Yield and Yield attributing characteristics					
	**CW(cm)**	**CL(cm)**	**NCPB**	**AWB(g)**	**TY (Kg/3.9 m^2)**	**TY(t/ha)**
AVT-1 GNB-23-38	0.77	2.33	26.01	13.59	2.22	5.54
AVT-1 GNB-23-41	1.12	2.59	29.72	18.32	2.62	6.55
AVT-1 GNB-23-47	0.81	2.37	24.77	16.92	2.23	5.59
AVT-1 GNB-23-20	0.91	2.42	26.64	13.79	2.39	5.97
AVT-1 GNB-23-26	1.08	2.51	28.55	15.85	2.46	6.15
**Mean**	0.94	2.44	27.14	15.69	2.39	5.96
**S.Em(±)**	0.04	0.02	0.54	0.02	0.03	0.07
**$CD_{0.05}$**	0.11	0.06	1.59	0.05	0.08	0.20
***A. nodosum* dose**						
0.5 ml/L	0.986	2.46	27.36	15.7	2.41	6.03
1 ml /L	0.996	2.47	27.68	16.09	2.43	6.06
1.5 ml/L	0.83	2.40	26.37	15.29	2.32	5.79
**Mean**	0.94	2.44	27.14	15.69	2.39	5.96
**S.Em(±)**	0.03	0.02	0.42	0.01	0.02	0.05
**$CD_{0.05}$**	0.08	0.05	NS	0.04	0.06	0.15

CW: Clove weight; CL: Clove length; NCPB:Number of cloves per bulb; AWB:Average weight of bulb; TY: Total Yield; Values with same letter(s) within a column are not significantly different at P= 0.05 (Duncun's Multiple Range Test, DMRT) . NS = Not Significant at P=0.05.

nge Test, DMRT) . NS = Not Significant at P=0.05.
